# The Market Access Society launches a new journal

**DOI:** 10.3402/jmahp.v1i0.21835

**Published:** 2013-08-06

**Authors:** Mondher Toumi

Welcome to the *Journal of Market Access and Health Policy*!

*The Journal of Market Access and Health Policy* (*JMAHP*) is the first peer-reviewed journal solely dedicated to Market Access. *JMAHP* is an Open Access Journal and the official organ of the Market Access Society (MAS). It aims at conveying transparency and consolidating knowledge around the market access research area, engaging in a forum of debate to support an international dialogue, with the overall goal to be the reference desk in market access around the world.

The primary objective of *JMAHP* is to consolidate the concept of “market access” as a multidisciplinary field spanning not only economic but also technical, scientific, sociological, psychological, and policy perspectives. *JMAHP* is in line with MAS's vision and mission to link all components that impact on “market access” to provide a convergent and broader perspective in a single specialized journal. The journal specifically encourages and welcomes studies from emerging countries in which changing markets and landscapes make market access seen as a significant challenge.

I'm honoured to take on the position of Editor-in-Chief of *JMAHP*. However, I could not have done this without the support of an outstanding editorial team who will not only guarantee the scientific quality of the submitted articles but also help me develop *JMAHP* to become a key resource for academics, regulators, industry, and decision makers. The journal has an editorial board of 25 excellent members with whom I'm delighted to work. We are eager together to develop this journal to serve as a reference desk for industry, regulators, and academics that in this way have free and immediate access to innovative research articles, discussion papers for best practices, and valuable guidelines. Each one of the editors are highly renowned colleagues representing their specific field of expertise and will work as ‘'ambassadors’’ for *JMAHP* around the world.

The main inspiration for developing *JMAHP* was to create a single forum to provide educational and the most up-to-date information in the fast evolving environment of “market access”, and to disseminate knowledge, new policies, experts’ opinions and expertise to support transparency around this field, and to share this with colleagues around the world who take an interest in market access and health policies. *JMAHP* will feature all levels of evidence-based research, guidance, policies, debates and hands-on information for our readers. In this way it is hoped that it will contribute to enhancing communication and transparency in the field.

Market access is the fundamental element of a product's commercial success. Its complexity is hard to examine, because of the multitude of rules in each different country. In addition, the healthcare market has recently seen changes in the way in which services are provided to patients: patients are more and more informed about their condition and possible treatments, mostly due to widespread use of Internet; patient associations have become more politically active and are able to lobby for public funding of (often costly) treatments that can heal their condition; patients need to prioritize one GP and/or primary care unit and hospital, otherwise they might miss reimbursement from the insurer; doctors are obliged to follow the prescription guidelines issued by a national Health Technology Assessment (HTA) body, and might have less freedom to choose treatments.

Despite a large proportion of medicines becoming cannibalized by generic products, the pharmaceutical market value continues to grow but at a slower pace. The continuous growth of healthcare expenditures and more specifically pharmaceutical expenditures has put healthcare insurance under unbearable pressure.

The late 1990's and early 2000's saw a large number of cost containment measures which, however, failed to control the expenditure. Thus, faced with rapidly growing health care expenditure, payers have put in place an increasing number of regulations, procedures and cost containment measures for pharmaceutical expenses, with two goals:

- keep expenditure within affordability limits/or a budget voted by the government
- ensure optimal usage of available resources (best health outcomes for money spent)

The pharmaceutical industry has to face those significant Market Access regulations and hurdles, while launching new products. As a result, getting marketing authorization for a drug is no longer sufficient to obtain Market Access for a medicine. On the other side, payers in the health care market always act as gate-keepers for market access. The payer audience is growing fast, in a heterogeneous way with diverse perspectives.

*At launch, JMAHP* publishes four articles; more are already in the pipeline. Susana Murteira and co-workers have contributed with a very useful article, ‘'Drug reformulations and repositioning in pharmaceutical industry and its impact on market access: reassessment of nomenclature” proposing a reassessment of current nomenclature of reformulations and repositioning of medicinal products to further allow a more robust analysis of the added value of these products. This is the first article in a series of three studying the impact of drug reformulations and repositioning on market access conditions (1). Erpur Adalsteinsson has written a highly interesting paper, “Benefits of probabilistic sensitivity analysis - a review of NICE decisions”, assessing the cost effectiveness of recent technology appraisals published by NICE and compared them with the actual NICE decision based on probabilistic sensitivity (2). You will also find a debate piece in two parts by Cécile Rémuzat, “New drug regulations in France: what are the impacts on market access?” reviewing the impact on market access and pharmaceutical industry of the new drug regulations in France (3, 4).

Articles in *JMAHP* will be freely available worldwide via www.jmahp.net without any need for registration or subscription. This will ensure maximum visibility and is completely in line with our vision to promote transparency and touch base with colleagues worldwide. Being an Open Access journal means that your article will reach researchers, payers, allied health care professionals, and patient groups, as well as policy makers, media and the general public from all parts of the world.

The publisher is currently working actively to have *JMAHP* indexed/tracked/covered by major indexing services such as PubMed Central/PubMed and MEDLINE, and will try to achieve indexing with Thomson Reuters Web of Knowledge as soon as the volume of content and other required criteria can be met. Material published by *JMAHP* will be distributed by EBSCO, one of the worlds’ largest content aggregators and research database providers.

To conclude, I invite you to submit an article to *JMAHP* on any of the topic areas that would contribute to the aims of the journal.

Finally, I would like to thank our team and the Co-Action Publishing staff for their continuous efforts and invaluable contribution to setting *JMAHP* in motion.

*Mondher Toumi* Editor-in-Chief
